# The African Teledermatology Project: Providing access to dermatologic care and education in sub-Saharan Africa

**Published:** 2009-11-19

**Authors:** Jennifer Weinberg, Steven Kaddu, Gerald Gabler, Carrie Kovarik

**Affiliations:** 1 Department of Dermatology, University of Pennsylvania School of Medicine,; 2 Department of Dermatology, Medical University of Graz, Austria,; 3 Department of IT and Telecommunications, Graz University Clinics and General Hospital, Austria

## Abstract

**Background:**

Telemedicine allows health providers in remote areas to transfer information for medical consultation anywhere in the world and serves to support local health workers through discussion and access to pertinent educational materials. Many developing nations have a dire shortage of doctors and other health resources. Therefore, affordable, easy-to-use technologies are imperative for providing care and much needed educational opportunities as well as reducing the limitations imposed by scarce resources.

**Methods:**

To identify the current extent of the Africa Teledermatology Project and key areas for improvements, an analysis of all consultations received to date was completed.

**Result:**

Between February 2007 and February 2009, 345 consultations from sites in thirteen Sub-Saharan African nations were received and processed via the project website. Although a wide range of mucocutaneous conditions were seen, the most frequent diagnoses included adverse drug reactions, atopic dermatitis and eczema, cutaneous infections, psoriasis and HIV/AIDS-related cutaneous diseases. Educational materials were designed to target these conditions.

**Conclusion:**

This research supports the value of store-and-forward teledermatology services for facilitating access to assistance in the diagnosis and management of cutaneous disease and increasing access to educational materials. The work demonstrates the feasibility and usefulness for a teledermatology network such as the African Teldermatology Project in improving the provision of care for skin diseases in sub-Saharan Africa. Additionally, this technology can be seen as a practical and effective manner to distribute information to local health workers with the hope of significantly improving their ability to recognize, diagnose and treat cutaneous conditions.

## Background

### Telemedicine and teledermatology

Telemedicine is defined as “the use of medical information exchanged from one site to another via electronic communications for the health and education of the patient or healthcare provider and for the purpose of patient care” by the American Telemedicine Association [1]. It merges medical expertise and communication technology to allow for patient examination, monitoring and management by a medical expert in a distant location, facilitating provision of clinical support services to remote, isolated and rural regions which lack access to higher level medical expertise [2]. Currently, the most common type of telemedicine is store-and-forward or asynchronous telemedicine, which allows patient history, exam findings and digital images to be transferred to a consulting physician who later evaluates the information and returns his impressions and recommendations [3]. As technology expands, mobile telephones are being employed for transfer of this data.

Teledermatology is the application of telemedicine to the specialty of dermatology and can serve as a means to provide support to local physicians and health care workers through discussion pertaining to diagnosis and management of patients with skin disease and access to pertinent educational materials [3]. Research suggests that when health care providers are trained to take appropriate photographs of the skin and provide relevant clinical information, the teledermatology encounter produces similar diagnoses as a live patient encounter with diagnostic agreement ranging from 81–89% and agreement on management decisions ranging from 90–96% in several studies [4,5,6,7]. Teledermatology is a safe and cost-effective manner for facilitating access to high-quality, cost-effective health care and can also play an essential role in clinician education, fostering increased understanding and independence in clinical practice while improving patient care [5,8,9,10]. Additionally, the use of teledermatology consultation as a second opinion service has been shown to be useful by allowing for discussion and/or confirmation of diagnosis as well as assistance with management decisions [11].

Many nations in the developing world have a dire shortage of doctors, especially specialists, such as dermatologists. Sub-Saharan Africa is home to some of the world’s most underdeveloped and resource-challenged countries with as few as 10 doctors per 100,000 inhabitants, and no dermatologists at all in many areas [12]. Due to the shortage of physicians, poor infrastructure and lack of resources, rural health care workers who serve the majority of the population in these areas are isolated from specialist support and current information. Therefore, affordable, easy-to-use technologies like teledermatology are imperative for providing patient care and much needed health care worker educational opportunities.

The 2001 World Health Organization report on the global burden of disease indicated that skin diseases were associated with annual mortality rates of 20,000 in Sub-Saharan Africa, comparable to mortality rates attributed to meningitis, hepatitis B, obstructed labor, and rheumatic heart disease in the same region [13]. Given the significant prevalence and morbidity of cutaneous disease and the fact that many systemic diseases, such as Human Immunodeficiency Virus (HIV)/Acquired Immunodeficiency syndrome (AIDS) and leprosy, have presenting skin manifestations, the need for effective diagnosis and treatment of skin conditions should be met A lack of basic knowledge of cutaneous manifestations and a shortage of elementary skills for skin disease management is often lacking at the primary care level [13,14]. In addition, skin diseases in the developing world are often transmissible but are readily treatable if properly recognized [15]. Teledermatology can facilitate access to high-quality, cost-effective health care and play an essential role in clinician education, fostering distribution of educational materials to the local workers in an effort to promote increased understanding and independence in clinical practice as well as improving patient care.

### The African Teledermatology Project

The African Teledermatology Project (http://africa.telederm.org) is an effort to utilize store-and-forward teledermatology technology to connect various medical institutions in Sub-Saharan Africa to more specialized dermatology units in the USA, Europe and Australia. The goal of the project is to create a network for dermatologic teleconsultation and e-learning, providing medical support to local clinicians throughout Africa. The consultation services allow for discussion pertaining to diagnosis and management of patients with skin diseases, links to educational resources, and access to a dermatologic curriculum created specifically for African sites in an effort to foster increased understanding and independence in clinical care and increase access to information and care in resource-limited areas.

In January 2007, the African Teledermatology Project was initiated through a collaboration between the Departments of Dermatology at the University of Pennsylvania, USA and the Medical University of Graz, Austria with additional collaboration from Mbarara University of Science and Technology and Makerere University in Uganda; the Penn-Botswana Program, Baylor International Pediatric AIDS Initiative and University of Botswana in Botswana, and The University of Queensland in Brisbane, Australia. Support has been provided by grants from the Austrian Academy of Science (“Kommission für Entwicklungsfragen der Österreichischen Akademie der Wissenschaften”) and the American Academy of Dermatology. Participating African medical centers who submit consultations to the site are located in Botswana, Eritrea, Kenya, Lesotho, Liberia, Malawi, Mozambique, Nigeria, Somalia, South Africa, Swaziland, Tanzania and Uganda. Sites submitting consultations must be equipped with the necessary hardware including digital cameras, computers with the appropriate software for case submission and processing and personnel who are trained to take appropriate photographs and upload the photographs and clinical information to the website.

The African Teledermatology Project utilizes the telederm.org platform, a free-access site which allows users to seek diagnostic and management advice from a group of experienced colleagues to allow for reciprocal exchange via an internet-based community [16]. The network allows users to register as clients or experts. Clients can submit cases via the internet to experts who review cases, write comments, suggest diagnoses, make management recommendations and/or discuss cases with other experts within the system. The web-based application allows store-and-forward medical cases to be uploaded with digital photographs and transferred via an internet connection to an expert teledermatologist for feedback and suggestions. Clients may choose to send a request for consultation to a selected expert or for discussion in an open forum. Automatic email notifications are sent to selected experts when a case is submitted for their review. Notifications are also sent via email to clients when a comment is posted to one of their cases. Expert answers and interactions remain in a private field for viewing only by the submitting client unless the case has been submitted for an open “discussion view” [16].

The project website also includes a curriculum with educational materials. These include a “discussion forum” for interesting or complex cases, exemplary cases with comprehensive discussions, a list of dermatology literature resources and a dermatology curriculum tailored for the most common conditions seen at participating sites. Additionally, detailed instructions are provided as to how to join the project as a consultant or submitting site.

## Methods

### Analysis

The current extent of the teledermatology program and potential areas for improvement were identified via analysis of the data received via the teledermatology consultation service. Consultations received to date were analyzed by chart review, noting: patient age and HIV status; healthcare provider(s) submitting the consultation and their location; primary and secondary clinical complaints; physical exam findings; the suspected diagnosis of the local submitting clinician; utilized and/or potential therapeutic interventions; completed tests and/or biopsies; the expert(s) replying to the consultation; the expert’s diagnosis and suggested management plan; outcome of consultation services; time for response to the consultation and number of photographs and/or pathological images submitted.

## Result

### Analysis Results

From the initiation of the Teledermatology Africa Project in February of 2007 to February 1, 2009, 345 consultations from sites in Sub-Saharan Africa have been received and processed via the project website. Of these tele-consultations, 46.24% (160) involved male patients and 41.55% (178) involved female patients (the sex of eight patients was unknown). Of the 174 with a known HIV-status, 71.84% were HIV-infected and/or had HIV/AIDS-associated skin conditions. The teleconsultation patients ranged in age from two weeks to 88 years, including 144 (41.62%) children <15 years of age, 63 (18.21%) young adults ages 16–30, 109 (31.50%) adults of middle-age (31–60 years), and 16 (4.62%) of adults over age 60.

### Development of an educational curriculum

Based on the analysis of consultations submitted to date, educational materials and health outreach modalities have been created. These educational modalities are intended to empower health providers while increasing independence in practice. Additionally, the information serves to familiarize participating consultants with commonly seen cutaneous conditions in sub-Saharan Africa using case examples and clinical photographs from local sites. PowerPoint ^®^ presentations detailing the prevalence, pathophysiology, morbidity and mortality, common presentations, diagnosis and management for dermatological disease seen frequently in sub-Saharan Africa were developed and posted under the “Curriculum” section of the Teledermatology Africa website. These materials introduce the reader to typical case examples, photographs of skin findings, techniques for diagnosis, typical pathological findings and locally available treatment options.

Over twenty topics are included in the educational curriculum. These include the diseases and findings seen most frequently in submitted teledermatology cases regarding the local patients. Educational materials covering adverse drug reactions, atopic dermatitis, bullous impetigo, candidiasis, cryptococcus, cutaneous T-cell lymphoma, dermatitis herpetiformis, dermatomyositis, discoid lupus, eosinophilic pustular folliculitis, epidermolysis bullosa, herpes simplex virus infection, Kaposi’s sarcoma, lichen planus, lymphogranuloma venereum, molluscum contagiosum, nutritionally-influenced skin conditions, pityriasis rosea, pigmentation changes, pruitic popular eruption, psoriasis, sarcoidosis, scabies, seborrheic dermatitis, tinea, varicella and warts are provided on the project’s website for all users to access. These topics are presented in a manner which familiarizes the reader with actual local presentations of disease processes seen in the sub-Saharan African region. Additionally informative presentations about how to perform common dermatologic procedures (punch and shave biopsies, cultures, potassium hydroxide preparations and Tzanck smears), how to take good clinical photographs for consultation submission and step-by-step instructions on joining the African Teledermatology Project as a consultant or a submitting site are provided.

## Discussion

Telemedicine has been shown to be an effective and accessible method for the diagnosis and management of many health conditions. This research supports the value of store-and-forward teledermatology services for facilitating access to assistance in the diagnosis and management of cutaneous disease and increasing access to educational materials for local providers. The work demonstrates the feasibility and usefulness for a broad teledermatology network such as the African Teldermatology Project in improving the provision of care for skin diseases for patients in sub-Saharan Africa. Additionally, this technology can be seen as a practical and effective manner to distribute information to local health care workers with the hope of significantly improving their ability to recognize, diagnose and treat cutaneous conditions.

Teledermatology has the potential to decrease health inequities especially in resource-limited settings. This technology provides an effective means not only to increase access to specialty medical services but also has the potential to increase local providers’ knowledge, and therefore independence in practice, over time as they are educated through the use of the consultation service and the feedback it provides. These benefits will expectantly increase the quality of health care provided to local patients in sub-Saharan Africa. Not only is teledermatology a useful forum for distribution of educational materials to underserved areas but it also has the potential to contribute to continuing medical education for both local and international health care providers, promote scientific collaboration and improve the cost-effectiveness and delivery of medical services.

Certain challenges have been identified in the current African Teledermatology Project. These include technical and system problems along with cultural and economic limitations. In a few of the consultations submitting to the site, a definitive diagnosis could not be reached secondary to poor image quality, insufficient clinical data, intrinsic case complications, difficulty appreciating three-dimensional qualities with the given technology and the loss of the information gained by hands-on patient examination. While some of these factors (i.e. inability to physically examine the patient) are inherent to the nature of store-and-forward telemedicine technology, others (i.e. poor image quality) have the potential to be improved through the provision of up-graded equipment and/or increased training of personnel in taking and submitting appropriate photographs and clinical information. To help address this aspect, materials detailing the proper technique for taking clinical photographs and successfully submitting consultations has been created and added to the project’s website.

Economic, cultural and technical limitations may arise when establishing such a program. Although skin conditions have been shown to occur in substantial frequency and inflict significant morbidity, care for cutaneous conditions is often overlooked in the allocation of health care resources. This is especially true in resource-limited settings already significantly strapped by epidemic levels of deadly disease such as HIV/AIDS which are often considered “more serious.” This lack of recognition has resulted in limited financial, technological and manpower resources at most health centers providing dermatological care. Even when external funding is available to establish teledermatology programs, implementation and ongoing utilization of these programs can be challenging, as the services place an increased burden on already scarce financial and human resources. Additionally, cultural beliefs and social structures can limit the implementation of teledermatology services. Some patients and providers express discomfort with the technology due to lack of trust in security or privacy concerns and may feel that the technology does not have potential to improve their health care experience. Potential ways to address these attitudes is through the provision of increased education about the technology and its potential benefits, provision of translation services to allow consultations to be submitted in the providers’ native language and assurance of adequate privacy and security. Additionally, utilizing local case examples and educating providers about the way targeted diseases manifest in populations similar to their own patient population can make the educational materials more relevant and accessible. Hope exists that not only can this technology directly address skin disease but that it can also improve the care of systemic diseases in which cutaneous findings are often the initial presentation.

## Conclusion

This research supports the value of store-and-forward teledermatology services for facilitating access to assistance in the diagnosis and management of cutaneous disease and increasing access to educational materials. The work demonstrates the feasibility and usefulness for a teledermatology network such as the African Teldermatology Project in improving the provision of care for skin diseases in sub-Saharan Africa. Additionally, this technology can be seen as a practical and effective manner to distribute information to local health workers with the hope of significantly improving their ability to recognize, diagnose and treat cutaneous conditions.
